# Quantifying intra-urban socio-economic and environmental vulnerability to extreme heat events in Johannesburg, South Africa

**DOI:** 10.1007/s00484-025-02971-y

**Published:** 2025-07-02

**Authors:** Craig Parker, Craig Mahlasi, Tamara Govindasamy, Lebohang Radebe, Nicholas Brian Brink, Christopher Jack, Madina Doumbia, Etienne Kouakou, Matthew Chersich, Guéladio Cissé, Sibusisiwe Makhanya, the HE2AT Center Group, Aakin Bobola, Abdoulaye Tall, Achiri Ndikum, Adaji Aishatu, Adja Ferdinand Vanga, Admire Chikandiwa, Akbar Waljee, Amy Beukes, Anna Steynor, Bonnie Joubert, Brama Kone, Bruce Hewitson, Cathy Mwangi, Cherlynn Dumbura, Chuansi Gao, Darshnika Lakhoo, Donrich Willem Thaldar, Duncan Mitchell, Dusty-Lee Donnelly, Elizabeth Frederick, Etienne Vos, Gciniwe Dlamini Baloyi, Gillian Marmelstein, Iba Dieudonné Dely, Ijeoma Solarin, Ilias Maliha, Jasper Maguma, Jetina Tsvaki, Ji Zhu, Khady Sall, Kimberly McAlpine, Kwesi Quagraine, Leon Mbano, Lisa Lois Harden, Lukman Abdulrauf, Margaret Brennan, Kimberly McAllister, Nontokozo Langa, Okoue Emerence, Olumuyiwa Adegun, Paul Ogendi, Peter Marsh, Peter Munyi, Pierre Kloppers, Piotr Wolski, Reneilwe Satekge, Rodger Duffett, Rutendo Sibanda, Ruvimbo Forget, Sabina Omar, Stanley Luchters, Tatenda Makanga

**Affiliations:** 1 Faculty of Health Sciences, Wits Planetary Health Research, University of the Witwatersrand, 7 York Road, Parktown, Johannesburg, 2193 South Africa; 2 IBM Research Africa, Johannesburg, South Africa; 3 Climate System Analysis Group, University of Cape Town, Cape Town, South Africa; 4 University Peleforo Gon Coulibaly, Korhogo, Côte d’Ivoire; 5 Centre Suisse de Recherches Scientifiques, Abidjan, Côte d’Ivoire

**Keywords:** Urban heat vulnerability, Spatial analysis, Healthcare access, Environmental justice, Climate adaptation, Principal component analysis, Johannesburg, Environmental health

## Abstract

Urban populations face increasing vulnerability to extreme heat events, particularly in rapidly urbanising Global South cities where environmental exposure intersects with socioeconomic inequality and limited healthcare access. This study quantifies heat vulnerability across Johannesburg, South Africa, by integrating high-resolution environmental data with socio-economic and health metrics across 135 urban wards. We examine how historical urban development patterns influence contemporary vulnerability distributions using principal component analysis and spatial statistics. Environmental indicators (Land Surface Temperature (LST), vegetation indices, and thermal field variance) were combined with socioeconomic and health variables (including indicators on crowded dwellings and healthcare access, self-reporting of chronic diseases) in a comprehensive vulnerability assessment. Principal Component Analysis revealed three primary dimensions explaining 56.6% (95% CI: 52.4–60.8%) of the total variance: urban heat exposure (31.5%), health status (12.8%), and socio-economic conditions (12.3%). Built-up areas showed weak but significant correlations with heat indices (ρ = 0.28, *p* < 0.01), while higher poverty levels demonstrated moderate positive correlations with LST (ρ = 0.41, *p* < 0.001). The spatial analysis identified significant clustering of vulnerability (Global Moran's I = 0.42, *p* < 0.001), with distinct high-vulnerability clusters in historically disadvantaged areas. Alexandra Township showed the highest vulnerability(HVI score: 0.87, LST: 29.8 °C ± 0.4 °C, NDVI: 0.08 ± 0.02), with factors characterising the high vulnerability in that area including limited healthcare access and extreme heat exposure. Northern suburbs formed a significant low-vulnerability cluster (Mean HVI = 0.23 ± 0.07, *p* < 0.001), benefiting from greater vegetation coverage and better healthcare access. These findings demonstrate how historical planning decisions continue to shape contemporary environmental health risks, with vulnerability concentrated in areas of limited healthcare access and high extreme heat exposure. Results suggest the need for targeted interventions that address both environmental and social dimensions of heat vulnerability, particularly focusing on expanding healthcare access in identified hotspots and implementing community-scale green infrastructure in high-risk areas. This study provides an evidence-based framework for prioritising heat-resilience initiatives in rapidly urbanising Global South cities while highlighting the importance of addressing historical inequities in urban adaptation planning.

**Supplementary Information:**

The online version contains supplementary material available at 10.1007/s00484-025-02971-y.

## Introduction

 Climate change is significantly reshaping urban life, with extreme heat events becoming more frequent and severe (IPCC Working Group, I 2022a; Ansah et al. 2024a). Urban populations face increasing vulnerability to these events, with risks shaped by complex interactions between environmental exposure, socioeconomic conditions, and health status (Estoque et al. 2023; Tuholske et al. 2021). This vulnerability is particularly acute in rapidly urbanising Global South cities, where historical inequalities and limited adaptive capacity compound environmental challenges (Tietjen et al. 2023; Abrahams 2019).

 Johannesburg, South Africa's largest city with 6.1 million inhabitants, presents a compelling case study of urban heat vulnerability (Souverijns et al. 2022). The city's rapid urbanisation, pronounced socio-economic inequalities, and historical legacy of apartheid urban planning create distinct patterns of environmental risk (Giombini and Thorn 2022a; Statistics South A, 2023). These factors interact with the urban heat island effect to produce heterogeneous vulnerability landscapes, particularly affecting disadvantaged populations (Schlosberg et al. 2022). Understanding these patterns is crucial for developing effective adaptation strategies, yet comprehensive analyses of urban heat vulnerability in African cities remain limited (Ansah et al. 2024a).

### Historical heat events and health impacts in Johannesburg

Johannesburg has experienced increasingly frequent and intense heat events in recent decades. The city reached its highest recorded temperature of 38 °C in January 2016 amid a severe heatwave and drought, breaking the previous record of 36.5 °C just weeks earlier in November 2015 (Strydom 2016). These extreme temperatures have tangible health consequences. Studies in South Africa's major cities, including Johannesburg, show that above a city-specific threshold (~ 18.7 °C apparent temperature for Johannesburg), all-cause mortality rises by ~ 0.9% per 1 °C increase, and by 2.1% per 1 °C among seniors 65 + (Wichmann 2017). Adverse heat-related health events and excess mortality often concentrate in the elderly, while morbidity increases across vulnerable groups (Arsad et al. 2022). Hospitals report more patients for dehydration, renal failure, heat stroke, and other heat-induced illnesses during extreme heat spells. Although official records counted only 11 direct heat-related deaths in South Africa in one recent assessment, researchers estimate the actual heat mortality burden to be much higher when considering excess deaths, suggesting significant underreporting of heat-related health impacts (Scovronick et al. 2018).

### Climate change projections for Johannesburg

Looking ahead, climate models project substantial warming for Johannesburg's region. Being an inland highland city, Johannesburg is expected to warm faster than global averages. By late-century (around 2100) under a high-emissions scenario, mean annual temperatures in interior South Africa could rise 6–7 °C above late 20th-century baselines, compared to a 3–4 °C increase in coastal areas (Wichmann 2017). Even mid-century projections are concerning: if global emissions remain high (RCP8.5), much of Johannesburg may warm by ~ 2 °C by 2050, dramatically increasing heat extremes (Wichmann 2017). One projection shows that the number of hot nights (minimum temperature > 20 °C) in Johannesburg will currently quadruple from about 10 per year to ~ 40 per year by 2050 – and up to 100 hot nights in the city's most heat-prone neighborhoods (World Bank Cities Support, P. 2024). Likewise, days over critical heat thresholds will become far more frequent. Researchers estimate an additional 3–4 weeks of very hot days per year in Johannesburg by mid-century, straining health systems and infrastructure (Wichmann 2017; Engelbrecht et al. 2015). These projected temperature increases, especially under unabated climate change, imply a greater risk of heat-related illness and mortality (Chersich 2023). The IPCC warns that beyond + 2 °C of global warming, heat-attributable mortality and morbidity in Africa will sharply rise (IPCC Working Group, I. 2022b). In Gauteng, this could translate to a doubling of heat-related deaths without adaptation by the end of the century (Garland et al. 2015).

### Apartheid urban planning and current vulnerability patterns

Johannesburg's socio-spatial layout – largely a legacy of apartheid-era planning – is pivotal in shaping heat vulnerability today. Historically, apartheid policies created wealthy, low-density suburbs with ample green spaces (often reserved for white residents) and dense townships with minimal vegetation or services for non-white residents (Giombini and Thorn 2022b). This has resulted in stark contrasts in urban heat exposure. Lush, tree-lined neighbourhoods like parts of Sandton enjoy cooler microclimates, while nearby townships such as Alexandra, with crowded tin-roof homes and few trees, register far higher temperatures (Souverijns et al. 2023). A recent citizen-mapping campaign confirmed that Johannesburg's hottest areas are overwhelmingly in formerly segregated townships, which can be approximately 6 °C hotter than the surrounding countryside on summer days (versus a 3–4 °C urban heat island in greener suburbs) (World Bank Cities Support, P. 2024; Souverijns et al. 2023; Habitat U.N., 2024). The lack of tree canopy and abundance of heat-absorbing surfaces in these areas directly stem from past planning inequities (World Bank Cities Support, P. 2024).

Moreover, housing quality differences exacerbate indoor heat exposure: informal housing, predominantly found in townships in South Africa (e.g., corrugated iron shacks), can become literal"ovens,"with indoor temperatures up to 15 °C higher than in modern housing during the day (Naicker et al. 2017). Residents of these neighbourhoods thus face a double burden – higher outdoor temperatures and more indoor heat retention – making them highly vulnerable during heatwaves. Apartheid geography has effectively hard-wired vulnerability into Johannesburg's landscape, clustering heat risk in the same marginalised communities that face economic and service deficits (Naicker et al. 2017). This explains why heat mortality and illness disproportionately affect poor, black communities in the city (Giombini and Thorn 2022c).

### Conceptual framework and current knowledge

 Urban heat vulnerability encompasses three interconnected dimensions: exposure, sensitivity, and adaptive capacity (IPCC Working Group, I 2022a). Exposure refers to the degree and duration of heat stress, typically quantified through temperature and environmental-related metrics and indices such as ambient air temperature above certain thresholds, Land Surface Temperature (LST), Urban Thermal Field Variance Index (UTFVI) and various human thermal discomfort indices. Heat metrics derived by integrating high spatial resolution satellite imagery and ground-based weather measurements provide high-resolution data on urban heat distribution (Wang 2023). Notably, dense urban areas with limited vegetation show significantly higher surface temperatures, with differences of up to 5 °C compared to well-vegetated neighbourhoods (Li et al. 2017; Oke 1982; Santamouris 2015; Voogt and Oke 2003; Zhou et al. 2014).

 Sensitivity reflects population susceptibility to heat stress, influenced by socio-economic conditions and health status. Recent studies demonstrate that chronic conditions such as cardiovascular disease, diabetes, and respiratory ailments significantly increase heat-related health risks (Watts, et al. 2023; Khosla et al. 2021; Desai et al. 2023). These health vulnerabilities intersect with socio-economic disparities in Johannesburg, compounding heat-health risks in disadvantaged communities (Souverijns et al. 2022; Urban Heat Study, G. 2023).

 Adaptive capacity, coping with and recovering from heat stress, depends heavily on access to healthcare, cooling infrastructure, and social support systems (World Health 2023). Limited healthcare access particularly affects heat vulnerability, as demonstrated by increased heat-related mortality in areas with restricted medical services (Ansah et al. 2024b). In Johannesburg, historical planning decisions continue to influence the distribution of these adaptive resources, creating persistent spatial patterns of vulnerability (Souverijns et al. 2022).

### Previous approaches to heat vulnerability assessment

Heat Vulnerability Indices (HVIs) have emerged as essential tools for identifying at-risk populations from extreme heat events. The development of HVIs traces back to early work by Cutter et al. on social vulnerability mapping and was further refined by Reid et al., who created one of the first national heat vulnerability indices in the United States (Cutter et al. 2003; Reid et al. 2009). Since then, three primary approaches to creating HVIs have emerged: equal-weighting methods where all variables contribute equally to the index; expert-weighting approaches where variables are weighted based on expert judgment; and statistical methods like Principal Component Analysis (PCA) that reduce dimensionality while objectively weighting variables based on their contribution to overall variance (Chow et al. 2012; Wolf and McGregor 2013; Zhu et al. 2014).

While HVIs have been widely applied in North America and Europe, comprehensive vulnerability assessments for African cities remain limited. Existing studies in Global South contexts have typically focused on environmental exposure (using satellite-derived temperature data) or social vulnerability, with few attempts to integrate both dimensions and health metrics (Niu et al. 2021). This gap is particularly significant given the unique vulnerability contexts of rapidly urbanising African cities, where historical inequalities, informal settlements, and limited adaptive capacity create distinct risk profiles.

Our PCA approach offers several advantages for the Johannesburg context: it reduces subjectivity in variable weighting, identifies latent vulnerability dimensions that may not be immediately apparent, and allows for integrating diverse data types (environmental, socioeconomic, and health) into a unified framework. This study builds on previous HVI work by combining high-resolution environmental data with ward-level socioeconomic and health metrics to create a spatially explicit vulnerability assessment at a scale relevant to municipal planning and intervention.

### Research gaps and study objectives

 While existing research has examined individual components of heat vulnerability, three critical gaps remain:

 Limited integration of environmental, socio-economic, and health data in vulnerability assessments, particularly in Global South contexts.

 Insufficient understanding of how historical urban development patterns influence contemporary heat vulnerability.

 Lack of high-resolution spatial analyses of heat vulnerability that can inform targeted intervention strategies.

 This study addresses these gaps through three specific objectives:

- Quantify the spatial distribution of heat vulnerability across Johannesburg by integrating high-resolution environmental data with socio-economic and health metrics

Analyse the relationship between historical urban development patterns and contemporary heat vulnerability.

- Identify priority areas for intervention based on compound vulnerability factors

## Data and methods

### Environmental data collection and processing

 We integrated environmental, socio-economic, and health data to assess heat vulnerability across Johannesburg's 135 wards. Environmental variables were derived from Landsat 8 Level 2, Collection 2 surface reflectance satellite imagery over path 170 and row 78 for the December-February 2020–2021 period. A maximum cloud cover of < 10% was specified (Giombini and Thorn 2022c). The data come readily corrected for atmospheric interference and calibrated to surface reflectance values. This selection yielded approximately one image per 16-day Landsat cycle, providing adequate temporal coverage while maintaining data quality. The thermal bands (10 and 11) were used to calculate LST using the split-window algorithm, which accounts for atmospheric effects by utilising the differential absorption between adjacent thermal infrared bands. This method, developed by Jiménez-Muñoz et al. (2014), incorporates atmospheric water vapour content derived from MODIS atmospheric profiles as an ancillary dataset to improve accuracy (Jiménez-Muñoz et al. 2014). Surface reflectance calibration had already been applied to the Collection 2 Level 2 data, eliminating the need for additional atmospheric correction for the optical bands.

Each derived index served a specific purpose in our assessment framework: LST provided a direct measure of surface heating, NDVI quantified vegetation density (a key mediating factor for urban heat), UTFVI measured relative heat intensity compared to the city average, and NDBI identified densely built-up areas prone to heat accumulation. All processing was performed using the Google Earth Engine platform with standardised algorithms for consistent analysis across the study area (Google Earth and Landsat 2021).

We acknowledge that LST has limitations as a proxy for human heat exposure. As noted by Muse et al. (2024), LST represents the temperature of urban surfaces (roads, roofs, vegetation) as observed from satellite overpass and does not directly measure the air temperature that people experience. The relationship between LST and actual surface air temperature (SAT) can vary by season, weather conditions, and urban context. While LST provides valuable information on relative spatial patterns of urban heat, it may not capture absolute exposure or the full diurnal cycle of heat. We justify our use of LST due to its high spatial resolution and ability to identify neighbourhood-scale thermal variations, which are crucial for our intra-urban vulnerability analysis while recognizing that it represents one component of heat exposure rather than a comprehensive measure.

### Urban Thermal Field Variance Index (UTFVI)

The Urban Thermal Field Variance Index (UTFVI) is a normalised measure that quantifies the intensity of urban heat island effects. It is calculated using the formula:

$$UTFVI\hspace{0.17em}=\hspace{0.17em}(Ts-Tmean)/Tmean$$

Where Ts is the LST at a specific location, and Tmean is the mean LST of the entire study area. This standardised approach allows for comparisons of relative heat intensity across different urban contexts. In our analysis, UTFVI values above 0.015 indicate areas with strong heat island effects, which often correspond to degraded urban thermal environments.

UTFVI offers several advantages as an environmental indicator of heat vulnerability. First, it normalises absolute temperature values relative to the city's average, highlighting anomalously hot areas regardless of the baseline temperature. Second, it provides a standardised classification scheme for thermal environmental quality: values below 0 indicate excellent or good conditions, values between 0–0.005 indicate normal conditions, values between 0.005–0.010 indicate poor conditions, values between 0.010–0.015 indicate bad conditions and values above 0.015 indicate worse conditions (Zhang 2006). Previous studies have associated these categories with ecological and human comfort evaluations (Al-Kafy 2021). In the Johannesburg context, UTFVI helps identify neighbourhoods experiencing disproportionate heat stress compared to the city average, which may indicate areas requiring prioritised intervention (Sheehan et al. 2021).

### LST as pragmatic choice for capturing intra-urban heat variability

While our environmental assessment utilised high-resolution (30 m) Landsat 8 data for intra-urban analysis, we acknowledge the resolution limitations of auxiliary climate data sources. ERA5 reanalysis data, with its horizontal resolution of approximately 31 km (~ 0.25°), was used only to provide broader climatic context and to identify extreme heat days at the city scale, not for intra-ward heat variability analysis. Although ERA5-Land offers enhanced resolution (9 km) and has shown improved performance in representing land surface processes compared to standard ERA5 (Muñoz-Sabater et al. 2021), even this resolution would be insufficient for capturing neighbourhood-scale thermal variations in a complex urban environment like Johannesburg[49]For this reason, our intra-urban heat exposure analysis relied primarily on the high-resolution Landsat data to derive the LST.

### Gauteng City-Region observatory quality of life survey

This study's socioeconomic and health data were derived from the Gauteng City-Region Observatory (GCRO) Quality of Life Survey 2020–2021 (Hamann and Kadt 2020). The GCRO is an independent research organisation established through a partnership between the University of Johannesburg, the University of the Witwatersrand, and the Gauteng Provincial Government. The Quality of Life survey is conducted biennially and serves as the most comprehensive assessment of living conditions, socioeconomic status, and well-being in the Gauteng province.

The 2020–2021 survey (6th iteration) employed a stratified random sampling approach designed to be statistically representative at the ward level. It involved 13,616 in-person household interviews across Gauteng, with 8,215 specifically in Johannesburg. The survey aimed to achieve approximately 60 respondents per ward, with a minimum threshold of 40 valid responses required for inclusion in our analysis. This approach resulted in comprehensive coverage of 135 of Johannesburg's 137 wards, with two excluded due to insufficient sample size.

It is important to note that the 2020–2021 survey coincided with the COVID-19 pandemic, which caused significant socioeconomic disruptions in Gauteng. To assess the impact of the pandemic on our vulnerability indicators, we conducted a comparative analysis with the pre-pandemic GCRO 2017/18 survey (see Supplementary Table S1) (Gauteng City-Region 2017). This analysis revealed that while absolute values of indicators changed substantially (e.g., unemployment increased from 3.76% to 18.67%), the spatial patterns of healthcare access variables maintained strong consistency (r > 0.8). Economic indicators showed weaker spatial correlations, but the overall patterns of relative vulnerability remained largely stable. These findings suggest that our vulnerability assessment captures both chronic structural inequalities and acute pandemic impacts, with healthcare access-related vulnerabilities proving particularly stable across time periods.

For each ward, we extracted 18 variables related to socioeconomic status, healthcare access, and health conditions. These ward-level aggregates were calculated using appropriate weighting to ensure representativeness, with confidence intervals typically ranging from ± 3.5% to ± 5.0% at the 95% confidence level. The survey's rigorous quality control process included data validation, consistency checks, and imputation for selected variables where appropriate (Hamann and Kadt 2020).

### Selection of indicators that characterise heat vulnerability

Our selection of indicators that characterise heat vulnerability followed established frameworks that conceptualize heat vulnerability as a function of exposure, sensitivity, and adaptive capacity (IPCC Working Group, I 2022a) (IPCC Working Group, I. 2022b). Each indicator was chosen based on empirical evidence linking it to heat-related health outcomes or adaptive responses:

#### Environmental exposure indicators

- Land Surface Temperature (LST): Directly measures the thermal environment and has been linked to heat-related morbidity and mortality (Heaviside et al. 2017).
- Normalized Difference Vegetation Index (NDVI): Quantifies vegetation coverage, which mitigates urban heat through evapotranspiration and shading (Santamouris 2020).
- Urban Thermal Field Variance Index (UTFVI): Identifies areas experiencing disproportionate heat stress relative to the city average (Zhang 2006).
- Normalized Difference Built-up Index (NDBI): Captures urban density, which influences heat retention and nocturnal cooling rates (Chen et al. 2006).

#### Sensitivity indicators

- Demographic composition: Age structure and population density affect heat vulnerability, with children and older adults at higher risk (Nori-Sarma et al. 2022).
- Chronic health conditions: Pre-existing conditions like diabetes, hypertension, and heart disease significantly increase susceptibility to heat stress (Watts et al. 2023).
- Housing quality: Crowded dwellings (> 2 persons per room) and informal housing materials reduce thermal comfort and increase indoor heat exposure (Wright et al. 2019).
- Economic vulnerability: Food insecurity and participation in school feeding programs as an indicator of households with limited financial resources for heat adaptation (Schuster et al. 2017).

#### Adaptive capacity indicators

- Healthcare access: Distance to healthcare facilities (> 1 km), reliance on public healthcare, and lack of medical insurance limit access to treatment during heat emergencies (Ansah et al. 2024c).
- Infrastructure access: Availability of basic services like piped water affects the ability to maintain hydration and cooling during heat events (Murage et al. 2020).

Healthcare accessibility and health indicators were both derived from the GCRO survey data (Kadt, et al. 2020). Healthcare accessibility was evaluated using three specific measures: (1) Distance to Healthcare Facilities, defined as the proportion of households within 1 km of a healthcare facility; (2) Public Healthcare Utilization Rates, capturing the percentage of residents reliant on public healthcare facilities; and (3) Medical Insurance Coverage, measuring residents'financial accessibility to healthcare services through private insurance. For health status assessment, we extracted the proportion of households reporting chronic conditions (diabetes, hypertension, heart disease) and recent experiences with infectious diseases (HIV, tuberculosis, COVID-19). These complementary measures allowed us to analyse both healthcare access barriers and underlying health vulnerabilities that may compound heat risk. All health-related data was self-reported at the household level and then aggregated to ward level with appropriate statistical weighting, providing a comprehensive spatial picture of health-related vulnerability factors across Johannesburg.

This comprehensive set of indicators ensures that all major dimensions of heat vulnerability are captured, allowing for a nuanced assessment of how different factors combine to create compound vulnerability profiles across Johannesburg's diverse neighbourhoods.

### Analytical methods

**Fig. 1:**
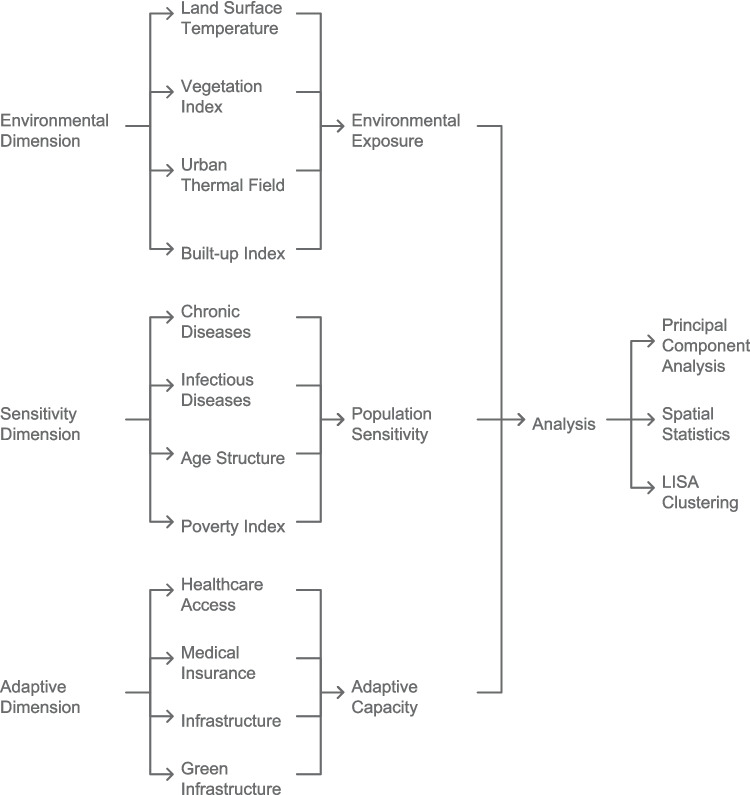
Analytical framework for assessing urban heat vulnerability in Johannesburg: Integration of environmental, social, and healthcare dimensions. Schematic showing the methodological approach and data integration process

We employed a three-stage analytical approach to assess heat vulnerability patterns across Johannesburg (Fig. 1). First, we prepared the data by conducting an initial assessment of completeness (finding no missing values across the 22 variables), performing z-score normalisation to ensure comparability, and harmonising spatial data at the ward level with appropriate weighting schemes.

Before implementing Geographically Weighted Principal Component Analysis (GWPCA), we conducted preliminary analyses to ensure statistical appropriateness. This included correlation analysis to identify potentially redundant indicators (examining variables with r > 0.9 and retaining moderately correlated variables [r = 0.6–0.9] that represented conceptually distinct vulnerability aspects), and tests for normality using Shapiro–Wilk tests, which revealed non-normal distributions for most variables (*p* < 0.05). Given these non-normal distributions, we used Spearman correlation coefficients between all variable pairs (α = 0.05) to avoid assumptions of linearity. The Kaiser–Meyer–Olkin measure of sampling adequacy (0.82) and Bartlett's test of sphericity (*p* < 0.001) confirmed the appropriateness of our dataset for GWPCA despite correlations among variables.

The GWPCA, using optimal bandwidth selection through cross-validation, generated local eigenvalues and eigenvectors for each ward, with components retained based on eigenvalue > 1 criterion. These components formed the basis of our Heat Vulnerability Index (HVI), weighted by explained variance and standardised to a 0–1 scale.

To address potential concerns regarding multicollinearity between LST and UTFVI (which is derived from LST), we conducted sensitivity analyses by creating three versions of our Heat Vulnerability Index: one with all variables (Original), one excluding LST (No_LST), and one excluding UTFVI (No_UTFVI). These sensitivity analyses helped verify the robustness of our vulnerability assessment approach (see Supplementary Materials, Sect. 9.3).

Spatial patterns were examined using Local Indicators of Spatial Association (LISA) analysis (Anselin 1995). We constructed a queen contiguity weights matrix and calculated Local Moran's I values for the HVI, with significance assessed through 999 *Monte* Carlo permutations and p-values adjusted for multiple testing. This approach identified statistically significant (*p* < 0.05) high and low vulnerability clusters and spatial outliers. All analyses were conducted in R (version 4.1.2) using spdep (1.1–8) and GWmodel (2.2–8) packages, with visualisations created using ggplot2 and tmap. Analysis scripts are available in supplementary materials.

The LISA analysis classified areas into four types: high-high clusters (vulnerable areas surrounded by other vulnerable areas), low-low clusters (resilient areas surrounded by other resilient areas), and two types of spatial outliers—high-low (vulnerable areas surrounded by resilient areas) and low–high (resilient areas surrounded by vulnerable areas).

## Results

### Overview of Urban heat vulnerability indicators

 The initial analysis of the 135 wards revealed substantial variations in environmental, socioeconomic, and health indicators across Johannesburg (Table 1). Key results from the vulnerability modelling and spatial analysis are presented in subsequent tables: Table 2 summarises the principal components underlying the Heat Vulnerability Index (HVI), Table 3 reports the LISA cluster types and their spatial characteristics, and Table 4 details the environmental, healthcare, and socio-economic profiles of high-vulnerability areas. Environmental metrics showed moderate variation, with LST ranging from 24.0 °C to 30.7 °C (mean = 27.9 °C ± 1.3 °C), while vegetation cover (NDVI) varied from 0.04 to 0.21, indicating significant differences in urban green space distribution Tables 2, 3 and 4

**Table 1:** Summary of environmental, socioeconomic, and health indicators across Johannesburg wards (N = 135)

Environmental Indicators	Mean ± SD	Min	25%	Median	75%	Max
Land Surface Temperature (°C)	27.92 ± 1.33	23.99	27.01	27.93	28.86	30.66
UTFVI	−0.06 ± 0.04	−0.18	−0.08	−0.06	−0.03	0.02
NDVI	0.14 ± 0.04	0.04	0.11	0.14	0.17	0.21
NDBI	0.35 ± 0.03	0.34	0.36	0.37	0.42	0.43
Socioeconomic Indicators	Mean ± SD	Min	25%	Median	75%	Max
Crowded Dwellings (%)	15.19 ± 11.97	0.00	9.49	15.01	23.73	84.62
Without Piped Water (%)	5.45 ± 9.45	0.00	0.00	1.89	5.67	62.95
Using Public Healthcare (%)	63.47 ± 30.49	3.56	31.75	73.69	87.80	98.55
Without Medical Insurance (%)	63.47 ± 26.44	7.07	38.85	73.59	84.62	97.11
At Risk of Hunger (%)	32.84 ± 21.91	0.00	9.49	36.78	49.73	70.01
School Feeding Schemes (%)	31.33 ± 21.32	0.00	10.49	33.72	49.32	79.63
Health Indicators	Mean ± SD	Min	25%	Median	75%	Max
Poor Health (%)	7.18 ± 4.48	0.00	3.97	6.09	9.95	19.98
Unable to Access Healthcare (%)	4.20 ± 3.67	0.00	1.50	3.83	5.58	16.97
Diabetes (%)	11.00 ± 5.00	0.00	7.00	11.00	15.00	35.00
Heart Disease (%)	5.00 ± 4.00	0.00	2.00	4.00	7.00	27.00
Hypertension (%)	23.00 ± 11.00	0.00	15.00	23.00	31.00	47.00
HIV (%)	8.00 ± 7.00	0.00	3.00	7.00	12.00	27.00
TB (%)	3.00 ± 4.00	0.00	0.00	2.00	5.00	12.00
COVID-19 (%)	4.00 ± 4.00	0.00	1.00	3.00	6.00	22.00

UTFVI = Urban Thermal Field Variance Index; NDVI = Normalized Difference Vegetation Index; NDBI = Normalized Difference Built-up Index. Values represent mean ± standard deviation or percentage where applicable

For Using Public Healthcare (%), standard deviation reflects the high variability between wards (30.49%) due to clustered access patterns. Twenty-five percent (25%) and seventy-five percent (75%) percentiles are shown as 25% and 75% respectively

**Table 2:** Principal component analysis results for heat vulnerability indicators

Component	Eigenvalue	Variance Explained (%)	Cumulative Variance (%)	Key Contributing Variables (Loading > 0.3)
PC1 (Urban Heat Exposure)	4.73	31.5	31.5	UTFVI (0.35), LST (0.34), NDBI (0.32), NDVI (−0.31)
PC2 (Health Status)	1.92	12.8	44.3	Chronic Diseases (−0.32), COVID-19 (−0.30), Healthcare Use (−0.22)
PC3 (Socio-economic)	1.85	12.3	56.6	Crowded Dwellings (−0.30), Household Hunger (−0.28)

Only loadings >|0.2| shown. Kaiser–Meyer–Olkin (KMO) measure = 0.82, Bartlett's test *p* < 0.001. PC = Principal Component

**Table 3:** LISA cluster analysis summary

Cluster Type	N (Wards)	Mean HVI	Moran's I	p-value	Key Characteristics
High-High	28	0.78 ± 0.09	0.68	< 0.001	LST > 29 °C, Limited Healthcare
Low-Low	35	0.23 ± 0.07	−0.55	< 0.001	High Vegetation, Good Healthcare
High-Low	12	0.65 ± 0.11	0.32	< 0.05	Informal Settlements
Low–High	8	0.35 ± 0.08	−0.28	< 0.05	Green Space Islands

HVI = Heat Vulnerability Index (0–1 scale), ± indicates standard deviation. All spatial statistics calculated using queen contiguity weights matrix

**Table 4:** Characteristics of high-vulnerability areas

Region	Environmental Indicators	Healthcare Access	Socio-Economic Factors	Health Indicators
Alexandra Township	LST: 29.8 °C ± 0.4 °C; NDVI: 0.08 ± 0.02	Public healthcare use: 89.2%; Avg distance to facilities: > 3 km	Household hunger risk: 70.0%; Crowded dwellings: 37.5%	Hypertension rate: 1.5 × avg; Chronic disease burden: High
Tembisa Areas	LST: 28.9 °C ± 0.5 °C; NDVI: 0.13 ± 0.03	Public healthcare use: 78.5%; Avg distance to facilities: 2.8 km	Household hunger risk: 56.4%; Crowded dwellings: 32.5%	COVID-19 rates: Above avg; TB prevalence: High
Tshepisong/Tshepiso	LST: 29.2 °C ± 0.3 °C; NDVI: 0.11 ± 0.03	Public healthcare use: 67.8%; Avg distance to facilities: 3.1 km	Household hunger risk: 58.4%; No piped water: 45.2%	Infectious disease: High; Healthcare access: Limited
Lenasia South	LST: 27.8 °C ± 0.6 °C; NDVI: 0.14–0.22	Public healthcare use: 64.5%; Avg distance to facilities: 2.5 km	Household hunger risk: 52.3%; Crowded dwellings: 26.8%	Chronic disease: High; Healthcare access: Limited
Soweto Clusters	LST: 27.4–29.6 °C; NDVI: 0.09–0.16	Public healthcare use: 75.8%; Avg distance to facilities: 2.9 km	Household hunger risk: 48.2%; Crowded dwellings: 28.4%	Chronic disease: Above avg; Healthcare access: Variable

Note: LST = Land Surface Temperature; NDVI = Normalized Difference Vegetation Index. All metrics shown with standard deviations where applicable

 Socioeconomic indicators demonstrated marked inequalities across wards. Access to basic infrastructure varied substantially: 94.5% of households across all wards had piped water, though in the most underserved wards, only 37.1% of households had access. Healthcare utilisation patterns showed similar disparities, with 63.47% (± 30.49%) of households within each ward relying on public healthcare facilities city-wide. Food insecurity was a significant concern, with 32.8% of households at risk of hunger across all wards, rising to over 70% in the most vulnerable areas. Housing conditions also reflected these inequalities, with 15.2% of households living in crowded conditions city-wide, though this reached up to 84.6% in the most densely populated wards.

Health indicators revealed substantial variation in household health challenges across wards. Hypertension prevalence varied across regions, ranging from 15% in the healthiest wards to 47% in the most affected areas (mean = 23.00% ± 11.00%). Diabetes showed similar geographic variation, ranging from 7 to 35% across wards (mean = 11.00% ± 5.00%). During the study period, COVID-19 affected different areas of the city unequally, with prevalence ranging from 1 to 22% across wards (mean = 4.00% ± 4.00%). These self-reported health conditions suggest significant disparities in health vulnerabilities across the city's communities, reflecting the potential influence of multiple comorbidities and underlying risk factors that vary across populations.

### Environmental-social correlations

 Examination of Spearman rank correlations revealed several notable associations between socioeconomic indicators and environmental exposures (Table 5). Vegetation cover (NDVI) showed significant negative correlations with household overcrowding (ρ = −0.56, *p* < 0.001) and food insecurity (ρ = −0.58, *p* < 0.001), suggesting a potential protective effect of green space. Conversely, LST was positively correlated with crowding (ρ = 0.29, *p* < 0.01), lack of health insurance (ρ = 0.30, *p* < 0.01), and pneumonia self-reporting (ρ = 0.26, *p* < 0.01), indicating a convergence of heat exposure and social vulnerability.

**Table 5:** Correlation matrix of key variables (Spearman's ρ)

Variable	LST	NDVI	Healthcare Access	Poverty Index
LST	1***	−0.29***	0.28**	0.41***
NDVI	−0.29***	1***	−0.65***	−0.56***
Healthcare Access	0.28**	−0.65***	1***	0.83***
Poverty Index	0.41***	−0.56***	0.83***	1***

Note: Significance levels *** *p* < 0.001, ** *p* < 0.01, * *p* < 0.05. N = 135 wards. LST = Land Surface Temperature; NDVI = Normalized Difference Vegetation Index

Thermal field variance (UTFVI), a measure of extreme heat exposure, was negatively associated with participation in school feeding programs (ρ = −0.33, *p* < 0.001). This suggests that higher heat stress areas may have unmet nutritional support needs. Strong intercorrelations were also observed among chronic health conditions, with pairwise Spearman coefficients ranging from 0.54 to 0.75 (*p* < 0.001) for diabetes, hypertension, and heart disease.

### Component analysis of vulnerability factors

Principal Component Analysis identified three significant components explaining 56.6% (95% CI: 52.4–60.8%) of total variance. The first component accounted for 31.5% of the variance (eigenvalue = 4.73), with the strongest loadings from environmental variables: UTFVI (0.35 ± 0.04), LST (0.34 ± 0.03), and negative loading from NDVI (−0.31 ± 0.03). The second component (12.8%) showed strongest loadings from health variables, while the third (12.3%) was dominated by socio-economic variables (Table 2).

**Fig. 2:**
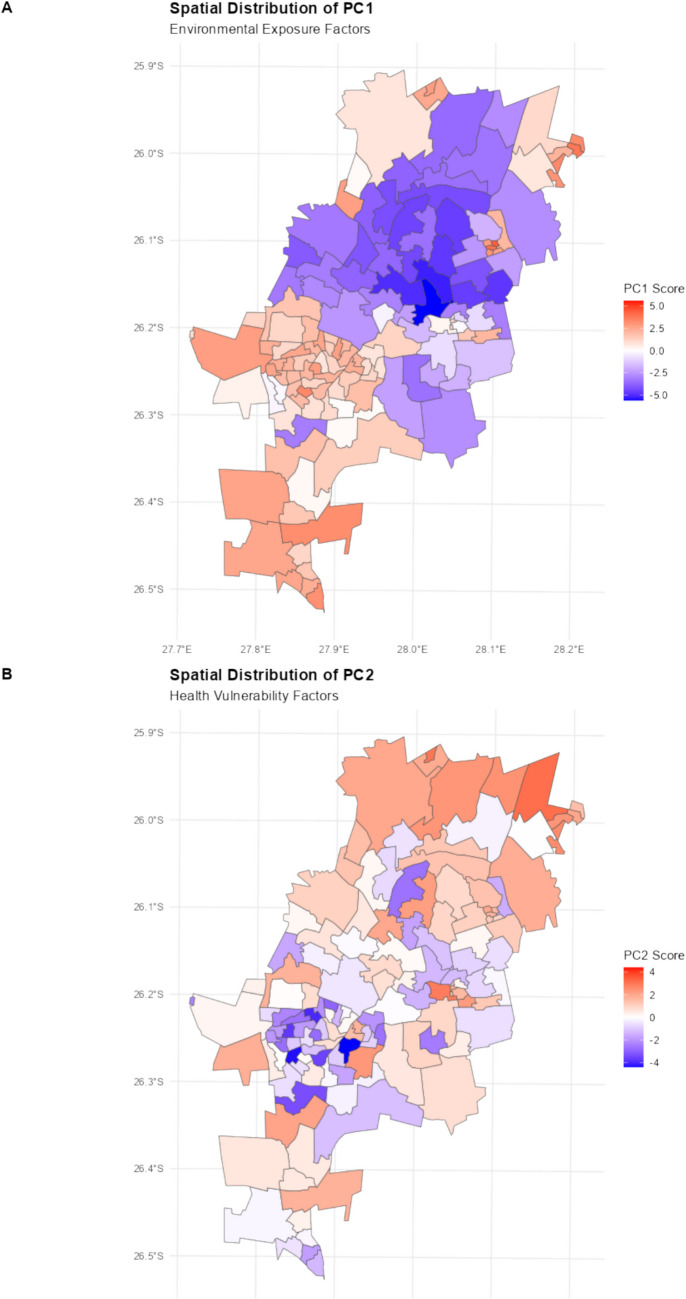
Spatial analysis of heat vulnerability components and index in Johannesburg. **a** Spatial distribution of the first principal component scores representing environmental exposure factors, with higher values in densely built areas; **b** Spatial distribution of the second principal component scores representing health vulnerability factors

Figure 2a presents the spatial distribution of the first principal component scores, which primarily captures environmental exposure factors. This component shows strong positive values in densely built areas with limited vegetation, particularly in the southern and northeastern regions. These areas are characterised by higher use of public healthcare, lower medical insurance coverage, higher household hunger risk, and higher LST. In contrast, negative scores (blue areas) represent more affluent regions with better environmental conditions that provide natural cooling and less heat stress, primarily in Johannesburg's central and northern parts.

The second component (Fig. 2b), dominated by health variables, shows a somewhat different spatial pattern, with concentrations in areas of lower socioeconomic status but not perfectly aligned with environmental exposure patterns. Negative scores (blue areas) indicate wards with higher prevalence of chronic diseases, higher proportions of elderly population, and greater overall disease burden, creating populations that are physiologically more vulnerable to heat stress. This health vulnerability shows a more dispersed pattern than environmental factors, with pockets of high vulnerability scattered throughout the city, while northern areas generally show lower health vulnerability.

This spatial variability between components highlights how different dimensions of vulnerability have distinct geographic distributions, underscoring the importance of a multidimensional approach to vulnerability assessment. Wards that display both poor environmental conditions and vulnerable health populations represent the highest risk areas requiring prioritized interventions that address both dimensions simultaneously.

**Fig. 3:**
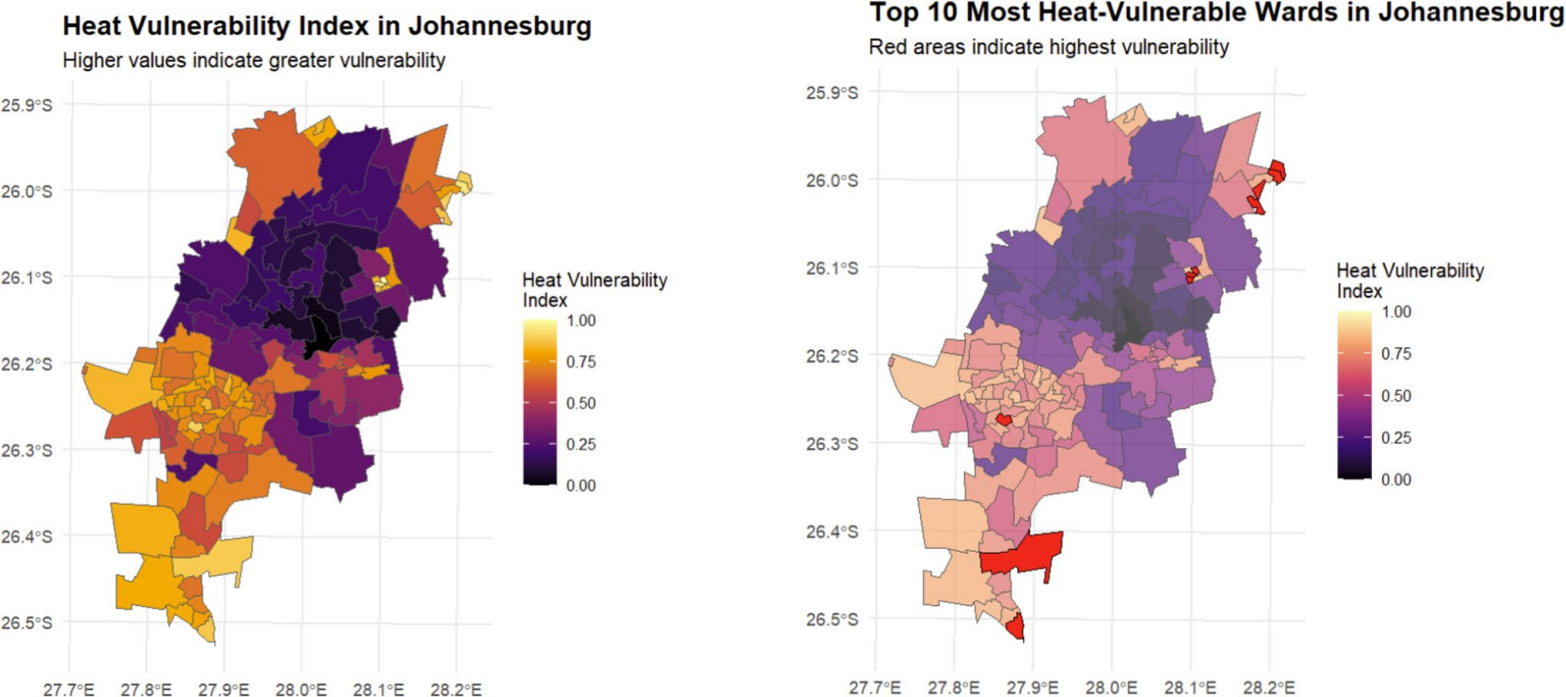
**a** Composite heat vulnerability index across all wards, with darker colors indicating higher vulnerability; **b** Detail of the ten most vulnerable wards, highlighting areas of concentrated risk. Ward boundaries are shown in black, and major roads are in grey

 We constructed a Heat Vulnerability Index (HVI) by combining these components, with each variable weighted according to its component loading and the proportion of variance explained by its respective component. The resulting index was standardised to a 0–1 scale. Figure 3a presents the spatial distribution of the HVI across Johannesburg, with darker colours indicating higher vulnerability. The map reveals a distinct north–south gradient in heat vulnerability, with concentrated areas of high vulnerability (orange) in the northeastern and southern regions of the city. A subset analysis of the ten most vulnerable wards (Fig. 3b) highlights particular hotspots in Alexandra Township and parts of Soweto, where all three component factors (environmental exposure, health status, and socio-economic conditions) contribute to elevated vulnerability scores.

 The HVI mapping reveals that the highest vulnerability scores (> 0.75) are concentrated in historically disadvantaged areas, particularly those with limited healthcare access and high environmental exposure. In contrast, the northern suburbs consistently show lower vulnerability scores (< 0.3), benefiting from greater vegetation cover and better healthcare infrastructure. This spatial pattern of vulnerability aligns with the component loadings from our PCA, demonstrating how environmental, health, and socio-economic factors combine to create distinct geographic risk patterns (Fig. 2).

### Spatial distribution of vulnerability

The LISA analysis revealed significant spatial clustering of heat vulnerability across Johannesburg (Global Moran's I = 0.42, *p* < 0.001). A summary of these cluster types and their defining characteristics is provided in Table 3. We identified 28 wards forming high-high clusters (Mean HVI = 0.78 ± 0.09, Moran's I = 0.68, *p* < 0.001), representing concentrated vulnerability hotspots. Conversely, 35 wards formed low-low clusters (Mean HVI = 0.23 ± 0.07, Moran's I = −0.55, *p* < 0.001), indicating areas of relative resilience. We also identified 20 spatial outliers, with 12 high-low outliers potentially indicating emerging hotspots and 8 low–high outliers suggesting localised resilience within otherwise vulnerable regions.

### Characteristics of high-vulnerability areas

**Fig. 4:**
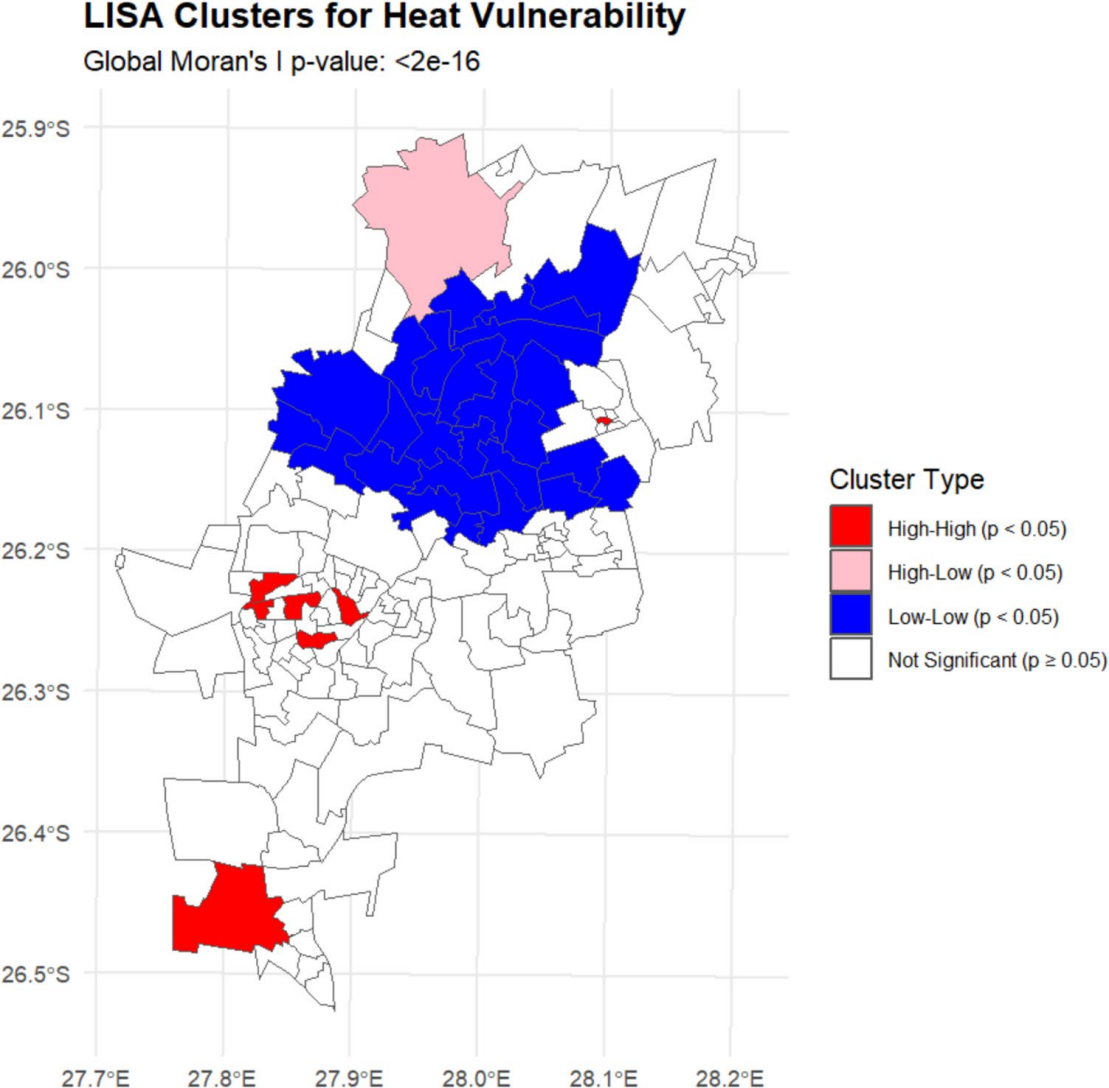
Spatial clustering of heat vulnerability in Johannesburg based on LISA analysis. High-high clusters are shown in red, low-low clusters are shown in blue, and spatial outliers are shown in purple. Statistical significance at *p* < 0.05

Within these spatial patterns, five distinct high-vulnerability clusters emerged, each representing unique combinations of environmental, healthcare, and socio-economic challenges (Table 4), (Fig. 4). Alexandra Township in the northeast emerged as the most vulnerable area, with extreme environmental exposure (LST: 29.8 °C ± 0.4 °C) combined with severely limited healthcare access. The Tembisa area showed intense urbanisation impacts (characterized by high built-up area index [NDBI > 0.40], low vegetation cover [NDVI < 0.10], and elevated land surface temperatureLST > 29 °C]), while Tshepisong/Tshepiso demonstrated how infrastructure deficits (defined as > 20% of households lacking access to one or more basic services including piped water, electricity, or proper sanitation) compound heat vulnerability. Notably, Lenasia South and Soweto clusters showed a different vulnerability pattern—while their LST readings were lower than other clusters (27.8 °C and 27.4–29.6 °C respectively), their vulnerability was driven by socioeconomic challenges including variable infrastructure quality and limited healthcare facility access, despite having relatively lower rates of public healthcare use and hunger risk compared to other high-vulnerability clusters. This variation in vulnerability drivers highlights the multidimensional nature of heat risk.

## Discussion

### Spatial patterns of heat vulnerability

 Our findings reveal how urban heat vulnerability in Johannesburg manifests through a complex interplay of environmental exposure, socio-economic conditions, and healthcare access. The stark spatial patterns we observed suggest that heat vulnerability is not merely a product of environmental factors but is deeply entwined with the city's historical development and ongoing socio-economic disparities.

 The emergence of five distinct vulnerability clusters, each with its unique combination of risk factors, challenges conventional approaches to heat adaptation in Global South cities. In Alexandra Township, for instance, we found that extreme environmental exposure (LST: 29.8 °C ± 0.4 °C) coincides with severely limited healthcare access, with nearly 90% of residents dependent on distant public facilities. This coupling of environmental and social vulnerability creates a heightened vulnerability that exceeds what either factor would suggest in isolation. Similar patterns emerge in Tembisa and Tshepisong, where limited infrastructure and high population density amplify the effects of elevated surface temperatures.

 Perhaps most striking is how these vulnerability patterns align with Johannesburg's historical spatial segregation. The strong statistical clustering we observed (Global Moran's I p-value: < 2e-16) reveals that heat vulnerability follows clear socio-spatial lines, with historically disadvantaged areas showing significantly higher risk profiles. This finding extends previous work on environmental justice in South African cities by quantifying how historical planning decisions continue to shape contemporary environmental health risks (Strauss 2019).

 The clear north–south divide in vulnerability patterns raises important questions about urban development and adaptation capacity. While northern suburbs benefit from abundant vegetation and robust healthcare infrastructure, creating significant low-vulnerability clusters, southern regions face compounded challenges. These lower vulnerability areas (defined quantitatively by HVI scores < 0.25, vegetation cover above the city median, medical insurance coverage > 60%, and household hunger risk < 15%) represent neighbourhoods with both environmental buffers against heat and socioeconomic resources that enhance adaptive capacity. This disparity suggests that conventional approaches to heat adaptation, which often focus solely on environmental interventions like urban greening, may be insufficient without parallel investments in social infrastructure and healthcare access.

 Our identification of spatial outliers—particularly high-vulnerability pockets within generally resilient areas—highlights the dynamic nature of urban heat risk. These anomalies often represent informal settlements or areas of rapid urban transformation, suggesting that vulnerability patterns are not static but evolve with urban development. This finding has important implications for adaptation planning, indicating the need for flexible, responsive interventions that can address emerging vulnerability hotspots.

### Environmental-social correlations and underlying mechanisms

 The correlations observed in the analysis reflect complex socio-spatial relationships in Johannesburg's urban landscape. The negative correlation between vegetation cover and overcrowding stems from historical urban development patterns where higher-density, lower-income areas were developed with minimal green space planning. Similarly, the positive correlation between LST and lack of health insurance represents the spatial concentration of disadvantage in Johannesburg, where areas of higher environmental stress often coincide with lower socioeconomic status. These are not necessarily causal relationships but manifestations of how historical socio-spatial segregation has created overlapping environmental and social vulnerabilities in specific city areas. The correlation between UTFVI and school feeding programs may indicate that areas experiencing the most intense urban heat effects have socio-economic profiles different from areas with high absolute temperatures but less extreme heat island effects.

### Implications for urban planning and heat adaptation

 These findings have significant implications for urban policy and planning. While targeted interventions in high-vulnerability clusters are needed, our results suggest that such interventions must simultaneously address multiple dimensions of vulnerability. Simple environmental modifications, while important, may only be sufficient with corresponding improvements in healthcare access and social infrastructure. The clear spatial patterns we observed provide a strong empirical basis for prioritising interventions while identifying compound risk factors, suggesting the need for integrated adaptation strategies.

 Our study also reveals several important areas for future research. The strong spatial clustering we observed suggests the need for a more detailed investigation of neighbourhood-level adaptation mechanisms, particularly in high-vulnerability areas. Additionally, identifying spatial outliers raises questions about the dynamics of vulnerability development and the potential for preventive interventions. Future work might also explore how these vulnerability patterns might shift under different climate change scenarios.

 These findings advance our understanding of urban heat vulnerability in several essential ways. First, they provide empirical evidence for the spatial clustering of heat risk in a major Global South city. Second, they demonstrate how historical planning decisions shape contemporary environmental health risks. Finally, they reveal the complex interactions between environmental exposure and social vulnerability that must be addressed in urban adaptation planning. As cities worldwide grapple with increasing heat stress under climate change, these insights offer valuable lessons for developing more equitable and effective adaptation strategies.

## Limitations

### Data limitations

 Although the GCRO Quality of Life Survey provides a rich dataset for exploring urban vulnerabilities, it has some constraints. The survey is not primarily designed as a health assessment, so the derived health metrics should be interpreted as general indicators rather than precise prevalence estimates. Additionally, the self-reported nature of the data may introduce biases, particularly for sensitive health topics.

### Multicollinearity assessment and variable sensitivity

A potential limitation is including both LST and UTFVI in our PCA, since UTFVI is derived from LST normalisation. We conducted sensitivity analyses with alternative HVI versions excluding either variable to address this. Results showed remarkable consistency (correlations > 0.99, stable PC1 variance of 31.46%−32.02%, and identical top 10 vulnerable wards across all versions). This confirms our vulnerability assessment is robust despite the correlation between these variables (see Supplementary Materials, Sect. 9.3).

### Spatial scale considerations

Another limitation concerns the spatial scale of our analysis. Using administrative wards as our unit of analysis provided practical advantages regarding data availability and policy relevance, but introduces potential modifiable areal unit problems (MAUP). Our analysis of local LST variability revealed that in most wards (94.8%), the local temperature variation was less than the global standard deviation across Johannesburg (1.33 °C), supporting our ward-lvel approach. However, 18.5% of wards exhibited substantial local variability with standard deviations exceeding 1 °C, with the highest reaching 2.03 °C.

This pattern was more pronounced in larger wards, with 23.9% of above-median sized wards showing high local variability (correlation between ward size and variability: r = 0.236). These findings suggest that while our ward-level analysis is appropriate for most of Johannesburg, some larger peripheral wards may contain significant internal temperature gradients that could obscure localized vulnerability hotspots. Future research could employ finer-scale analysis using dasymetric mapping or grid-based approaches to address this limitation, particularly in identified hotspot areas.

### Generalizability and future research directions

A notable contextual limitation is that data collection occurred during the COVID-19 pandemic. As detailed in Sect. 2.4 and Supplementary Table S1, our comparative analysis with pre-pandemic data showed that while absolute values of socioeconomic indicators changed substantially, spatial patterns of healthcare vulnerability maintained strong consistency (r > 0.8). This suggests our findings represent enduring structural patterns rather than solely pandemic effects, though acute pandemic impacts likely intensified existing vulnerabilities in the most affected communities.

 Our focus on Johannesburg offers a detailed case study but may limit the generalizability of the findings to other urban contexts. Cities vary in their specific environmental challenges, socioeconomic conditions, and governance structures, so the patterns observed here may not be universally applicable. Comparative studies across multiple cities would help assess the transferability of our vulnerability assessment approach and identify common themes and context-specific differences. Finally, while we examined a broad set of vulnerability indicators, additional factors that influence heat risk are likely not captured in our analysis. For example, we did not have access to high-resolution data on housing quality, social capital, or behavioural adaptations, which could all shape resilience to heat stress. Integrating a wider range of data sources and vulnerability proxies could provide an even more comprehensive picture.

 Despite these limitations, our study demonstrates the value of integrating environmental, socioeconomic, and health data to map urban vulnerability. Identifying key correlations and spatial patterns highlights potential intervention points and future research directions to enhance urban resilience. As cities grapple with the challenges posed by climate change, such interdisciplinary vulnerability assessments will be crucial for guiding equitable adaptation efforts.

## Conclusions

 This study provides a comprehensive spatial analysis of heat vulnerability in Johannesburg, revealing how environmental exposure and social inequality create distinct geographic risk patterns. Integrating multiple data streams and advanced spatial statistics, we identified five geographically distinct high-vulnerability clusters, each characterised by unique combinations of environmental exposure, healthcare limitations, and socioeconomic challenges (Global Moran's I p-value: < 2e-16). These vulnerability patterns demonstrate how historical planning decisions shape contemporary environmental health challenges in South African cities.

 Our findings challenge simplistic approaches to urban heat adaptation. While environmental factors account for the largest variance (31.5%), health access and socio-economic conditions play crucial mediating roles. While acknowledging potential within-ward variations, the spatial precision of our analysis, examining 135 wards across Johannesburg, provides actionable intelligence for urban planning and public health interventions. In identified hotspots like Alexandra Township and Tembisa, extreme surface temperatures combined with severely limited healthcare access create a compound vulnerability that neither factor would predict.

The strong correlations between environmental and socioeconomic factors reflect deep-rooted socio-spatial patterns requiring coordinated intervention strategies. As cities worldwide confront increasing heat stress due to climate change, our findings suggest that effective adaptation requires simultaneously addressing environmental exposure and social vulnerability. Future research should examine how these vulnerability patterns evolve and evaluate intervention effectiveness. Most crucially, our results indicate that climate change may exacerbate existing urban inequalities without concerted efforts to address both environmental and social dimensions of heat vulnerability.

## Supplementary Information

Below is the link to the electronic supplementary material.

Supplementary file1 (DOCX 1165 KB)

## Data Availability

The datasets analysed for this study are available from the following sources: -Environmental metrics were derived from ERA5 reanalysis data and Landsat 8 satellite imagery (December-February 2020–2021), available from the United States Geological Survey Earth Explorer. -Socio-economic and health data were obtained from the Gauteng City-Region Observatory (GCRO) Quality of Life Survey 2020–2021, publicly available at Gcro. -Analysis scripts for comparing GCRO data between survey periods are available at https://github.com/Logic06183/GCRO - Complete analysis scripts, processed data, and visualisation code are available at https://github.com/Logic06183/data_sources -Additional analysis files and supplementary materials are available from the corresponding author upon request.
