# Publisher Correction to: Quantifying intra-urban socio-economic and environmental vulnerability to extreme heat events in Johannesburg, South Africa

**DOI:** 10.1007/s00484-025-02988-3

**Published:** 2025-08-28

**Authors:** Craig Parker, Craig Mahlasi, Tamara Govindasamy, Lebohang Radebe, Nicholas Brian Brink, Christopher Jack, Madina Doumbia, Etienne Kouakou, Matthew Chersich, Guéladio Cissé, Sibusisiwe Makhanya, the HE2AT Center Group, Aakin Bobola, Abdoulaye Tall, Achiri Ndikum, Adaji Aishatu, Adja Ferdinand Vanga, Admire Chikandiwa, Akbar Waljee, Amy Beukes, Anna Steynor, Bonnie Joubert, Brama Kone, Bruce Hewitson, Cathy Mwangi, Cherlynn Dumbura, Chuansi Gao, Darshnika Lakhoo, Donrich Willem Thaldar, Duncan Mitchell, Dusty-Lee Donnelly, Elizabeth Frederick, Etienne Vos, Gciniwe Dlamini Baloyi, Gillian Marmelstein, Iba Dieudonné Dely, Ijeoma Solarin, Ilias Maliha, Jasper Maguma, Jetina Tsvaki, Ji Zhu, Khady Sall, Kimberly McAlpine, Kwesi Quagraine, Leon Mbano, Lisa Lois Harden, Lukman Abdulrauf, Margaret Brennan, Kimberly McAllister, Nontokozo Langa, Okoue Emerence, Olumuyiwa Adegun, Paul Ogendi, Peter Marsh, Peter Munyi, Pierre Kloppers, Piotr Wolski, Reneilwe Satekge, Rodger Duffett, Rutendo Sibanda, Ruvimbo Forget, Sabina Omar, Stanley Luchters, Tatenda Makanga

**Affiliations:** 1 Faculty of Health Sciences, Wits Planetary Health Research, University of the Witwatersrand, 7 York Road, Parktown, Johannesburg, 2193 South Africa; 2 IBM Research Africa, Johannesburg, South Africa; 3 Climate System Analysis Group, University of Cape Town, Cape Town, South Africa; 4 University Peleforo Gon Coulibaly, Korhogo, Côte d’Ivoire; 5 Centre Suisse de Recherches Scientifiques, Abidjan, Côte d’Ivoire

**Publisher Correction to: International Journal of Biometeorology**

10.1007/s00484-025-02971-y

In the originally published version of this article, Aakin Bobola, was mistakenly listed as an author. This was an error introduced during typesetting and has since been corrected. The correct list of authors is:

Craig Parker, Craig Mahlasi, Tamara Govindasamy, Lebohang Radebe, Nicholas Brian Brink, Christopher Jack, Madina Doumbia, Etienne Kouakou, Matthew Chersich, Guéladio Cissé, and Sibusisiwe Makhanya for the HE2AT Center Group

Springer apologizes for the oversight and any confusion this may have caused. The original article has been corrected.

