# Supplementary material for: Quantifying intra-urban socio-economic and environmental vulnerability to extreme heat events in Johannesburg, South Africa
Source: Int J Biometeorol. 2025 Jul 2;69(10):2501–17. doi: 10.1007/s00484-025-02971-y (PMC12540561; doi:10.1007/s00484-025-02971-y)

## Supplementary materials

### **Table S1:Comparative Analysis of Key Socioeconomic and Healthcare Indicators Between GCRO Quality of Life Surveys (2017-2018 and 2020-2021)**

| **Indicator** |  | **2017-2018 (%)** | **2020-2021 (%)** | **Absolute Change (pp)** | **Relative Change (%)** | **Spatial Correlation** | **Rank Correlation** |
| --- | --- | --- | --- | --- | --- | --- | --- |
| Unemployment |  | 3.76 | 18.67 | 14.91 | 396.5 | 0.044 (Very Weak) | 0.086 (Very Weak) |
| Food Insecurity |  | 7.13 | 61.69 | 54.56 | 765.2 | 0.582 (Moderate) | 0.612 (Moderate) |
| No Medical Insurance |  | 11.36 | 31.14 | 19.78 | 174.1 | 0.397 (Weak) | 0.440 (Weak) |
| Public Healthcare Use |  | 23.11 | 75.42 | 52.31 | 226.4 | 0.891 (Strong) | 0.760 (Strong) |
| Informal Dwellings |  | 62.57 | 11.23 | -51.34 | -82.1 | 0.815 (Strong) | 0.768 (Strong) |

Notes:

- pp = percentage points
- Spatial Correlation: Pearson correlation coefficient between ward-level values across surveys
- Rank Correlation: Spearman correlation coefficient measuring stability of ward rankings
- Interpretation of correlation strength: Very Weak (<0.2), Weak (0.2-0.4), Moderate (0.4-0.6), Strong (0.6-0.8), Very Strong (>0.8)
- Data source: GCRO Quality of Life Surveys 2017-2018 (v1.1) and 2020-2021 (new-weights-v1)

### **Table S2: Comparative Rankings of Combined Heat Vulnerability Index (HVI) and Component Factors**

| **Rank** | **Heat Vulnerability Index (HVI) Value Ward** | **Heat Vulnerability Index (HVI) Value Value** | **Heat Exposure Ward** | **Heat Exposure Value** | **Low Vegetation Ward** | **Low Vegetation Value** | **Public HC Ward** | **Public HC Value** | **No Insurance Ward** | **No Insurance Value** | **Hunger Risk Ward** | **Hunger Risk Value** | **Crowded Ward** | **Crowded Value** |
| --- | --- | --- | --- | --- | --- | --- | --- | --- | --- | --- | --- | --- | --- | --- |
| 0 | Ward 87 | 1 | Ward 116 | 30,66 | Ward 63 | 0,045 | Ward 128 | 98,6 | Ward 128 | 97,1 | Ward 2 | 70 | Ward 113 | 51,5 |
| 1 | Ward 117 | 0,97 | Ward 108 | 30,56 | Ward 116 | 0,054 | Ward 35 | 96,5 | Ward 61 | 94,5 | Ward 6 | 69 | Ward 95 | 43,4 |
| 2 | Ward 88 | 0,94 | Ward 113 | 30,37 | Ward 108 | 0,057 | Ward 127 | 95,6 | Ward 116 | 94,2 | Ward 48 | 66,7 | Ward 114 | 40,8 |
| 3 | Ward 72 | 0,92 | Ward 135 | 30,37 | Ward 62 | 0,058 | Ward 21 | 94,8 | Ward 19 | 94,2 | Ward 121 | 64,4 | Ward 37 | 40,5 |
| 4 | Ward 90 | 0,92 | Ward 8 | 30,35 | Ward 133 | 0,065 | Ward 40 | 94,4 | Ward 111 | 92,9 | Ward 130 | 63,7 | Ward 107 | 40 |
| 5 | Ward 73 | 0,91 | Ward 107 | 30,14 | Ward 107 | 0,068 | Ward 24 | 93,5 | Ward 6 | 91,6 | Ward 15 | 63,2 | Ward 2 | 37,5 |
| 6 | Ward 103 | 0,9 | Ward 96 | 30,09 | Ward 77 | 0,075 | Ward 130 | 93,3 | Ward 75 | 91,6 | Ward 45 | 61,5 | Ward 75 | 37,2 |
| 7 | Ward 104 | 0,89 | Ward 32 | 30,06 | Ward 79 | 0,076 | Ward 50 | 93,3 | Ward 53 | 91,2 | Ward 47 | 61,3 | Ward 116 | 36 |
| 8 | Ward 99 | 0,88 | Ward 105 | 29,98 | Ward 76 | 0,076 | Ward 34 | 92,2 | Ward 59 | 91 | Ward 51 | 60,2 | Ward 35 | 35,7 |
| 9 | Ward 106 | 0,87 | Ward 76 | 29,88 | Ward 75 | 0,078 | Ward 11 | 92,1 | Ward 127 | 90,8 | Ward 41 | 60,1 | Ward 44 | 33,2 |

This table illustrates disparities between the Combined Heat Vulnerability Index (HVI) and its component factors (Heat Exposure, Vegetation, Healthcare Access, and Socio-Economic Conditions). Highlighting these differences underscores the multidimensional nature of heat vulnerability and the need for targeted interventions addressing both individual drivers and their combined impacts.

- - - 1. Supplementary Code and Data Repositories
      2. To promote transparency and reproducibility, we have made all analysis code and data processing scripts publicly available in the following repositories:
      3. 1. GCRO Survey Comparison Analysis: <https://github.com/Logic06183/GCRO>

- Contains scripts used to analyse and compare the 2017-2018 and 2020-2021 GCRO Quality of Life Surveys

- - - 1. - Includes code for calculating correlations, generating visualisations, and quantifying pandemic impacts on key variables
      2. 2. Main Analysis Scripts and Data Sources: <https://github.com/Logic06183/data_sources>

- Contains the complete analysis pipeline used in this study

- - - 1. - Includes scripts for processing environmental data, calculating vulnerability indices, and performing spatial analyses
      2. - Contains visualisation code for generating all figures in the manuscript
      3. - Provides reproducible R scripts for Principal Component Analysis and LISA cluster analysis
      4. We encourage interested researchers to use these resources for validation, extension, or application to other urban contexts.

### **Sensitivity Analysis for LST and UTFVI Variables**

To address potential multicollinearity concerns between Land Surface Temperature (LST) and Urban Thermal Field Variance Index (UTFVI), we performed a sensitivity analysis examining how their inclusion or exclusion affects the Heat Vulnerability Index. The correlation between LST and UTFVI was very high (r = 0.995), confirming the need for this analysis.

We created three versions of the Heat Vulnerability Index:

1. Original: Including all 22 variables
2. No_LST: Excluding LST (21 variables)
3. No_UTFVI: Excluding UTFVI (21 variables)

Principal Component Analysis was performed for each version, and the results are summarized in Table S3.

**Table S3: Sensitivity Analysis Results for Different HVI Versions**

| **Analysis Version** | **Variables** | **PC1 Variance (%)** | **Mean HVI** | **SD HVI** |
| --- | --- | --- | --- | --- |
| Original | 22 | 31.46 | 0.5606 | 0.2632 |
| No_LST | 21 | 31.75 | 0.5689 | 0.2777 |
| No_UTFVI | 21 | 32.02 | 0.5686 | 0.2758 |

The correlations between the three HVI versions were exceptionally high:

- Original vs. No_LST: r = 0.997
- Original vs. No_UTFVI: r = 0.997
- No_LST vs. No_UTFVI: r = 0.999

Most importantly, all three indices identified exactly the same wards in the top 10 most vulnerable areas, with only minor differences in their rankings. This high level of consistency demonstrates that our vulnerability assessment approach is robust to the inclusion or exclusion of either LST or UTFVI, despite their high correlation. These findings support the validity of our original analysis and indicate that multicollinearity between these two variables does not significantly impact the identification of vulnerability hotspots across Johannesburg.


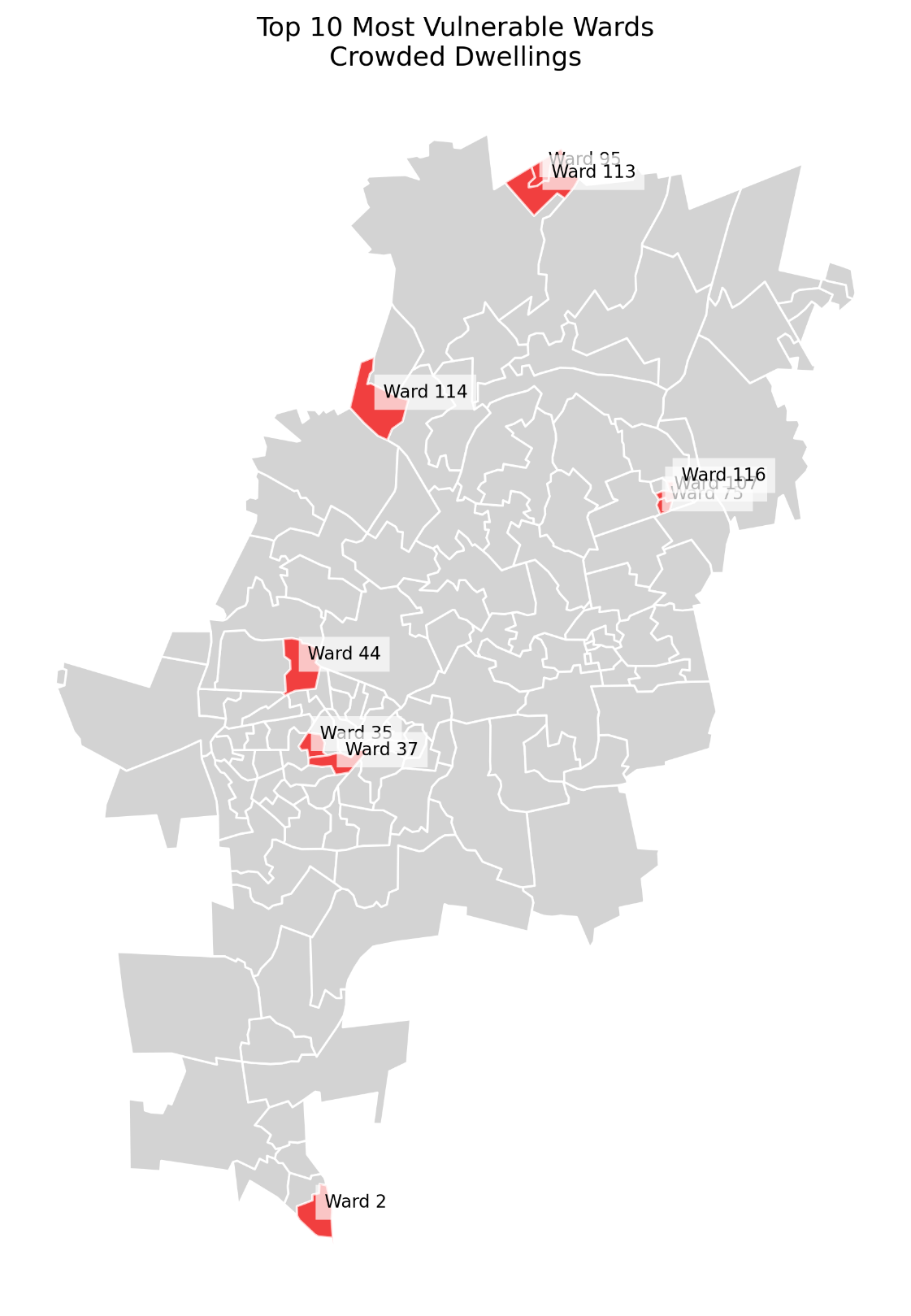

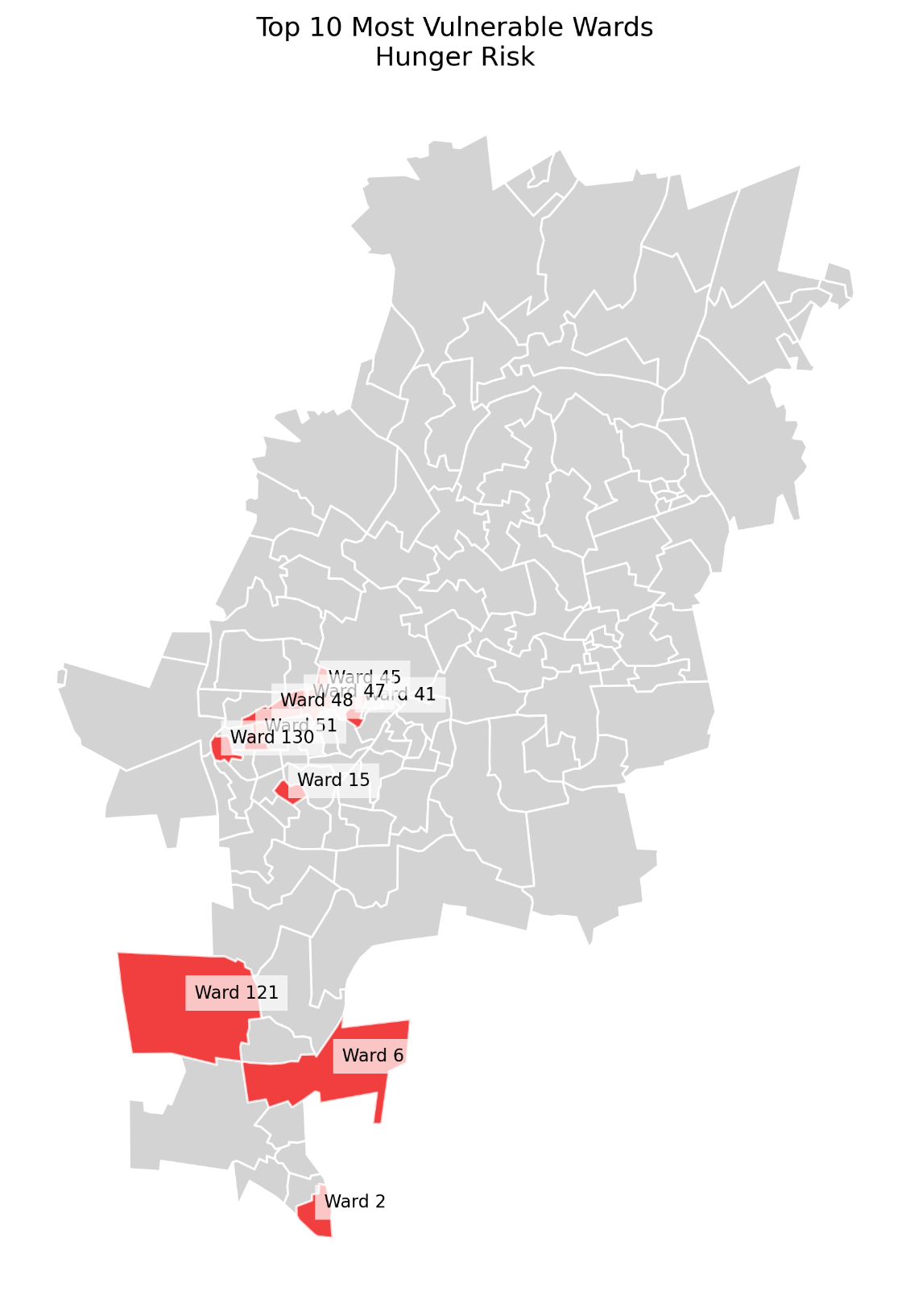

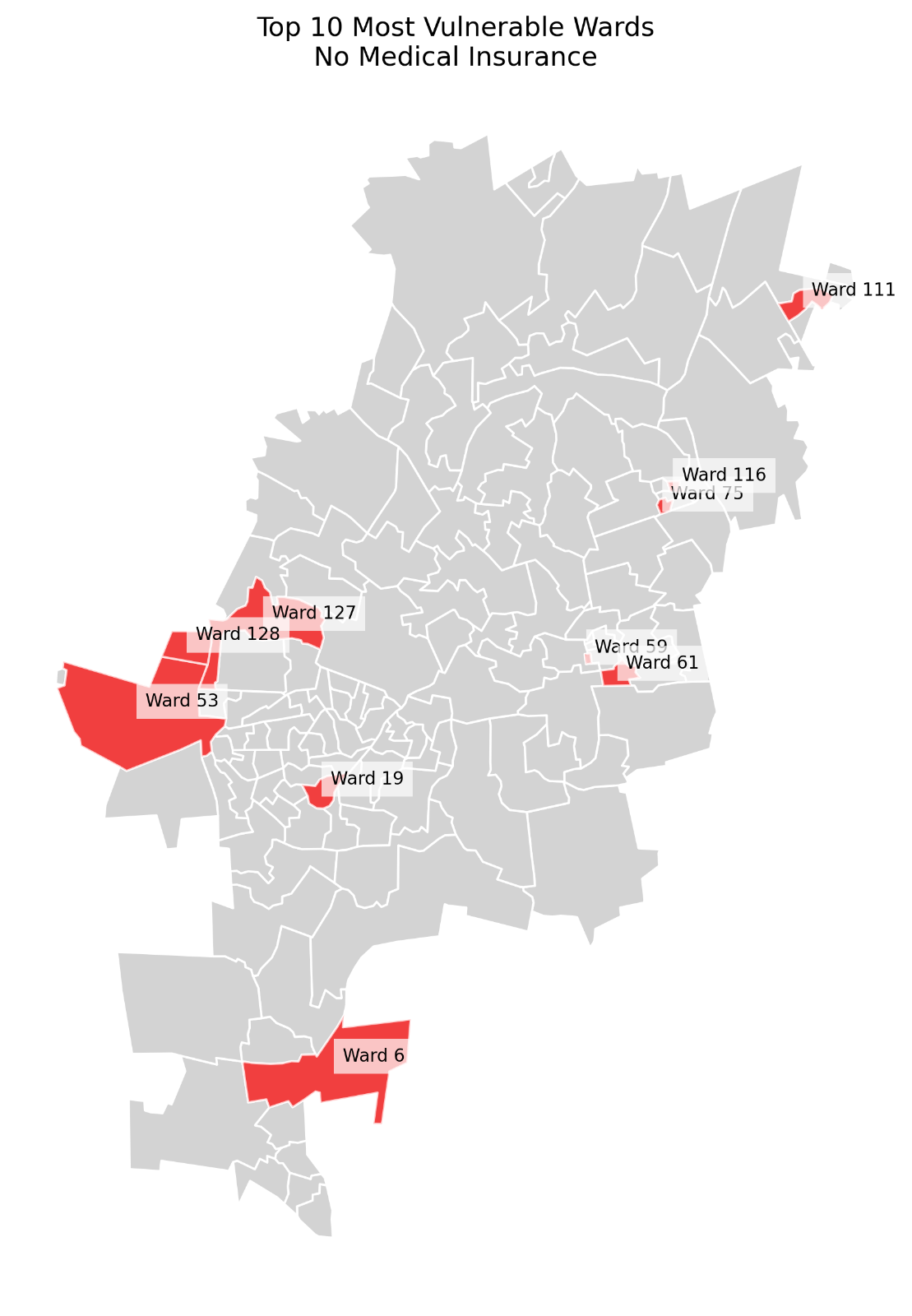

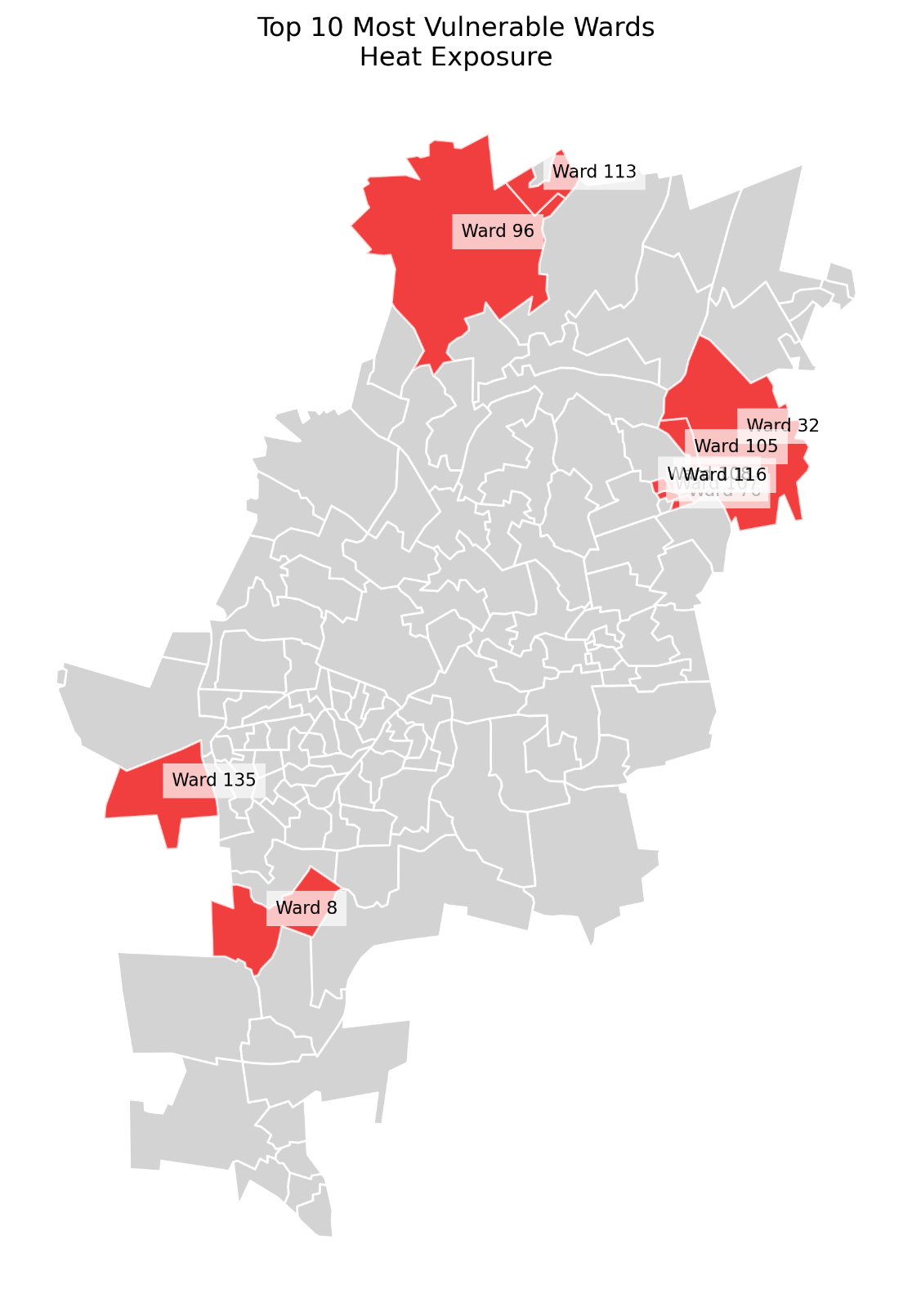

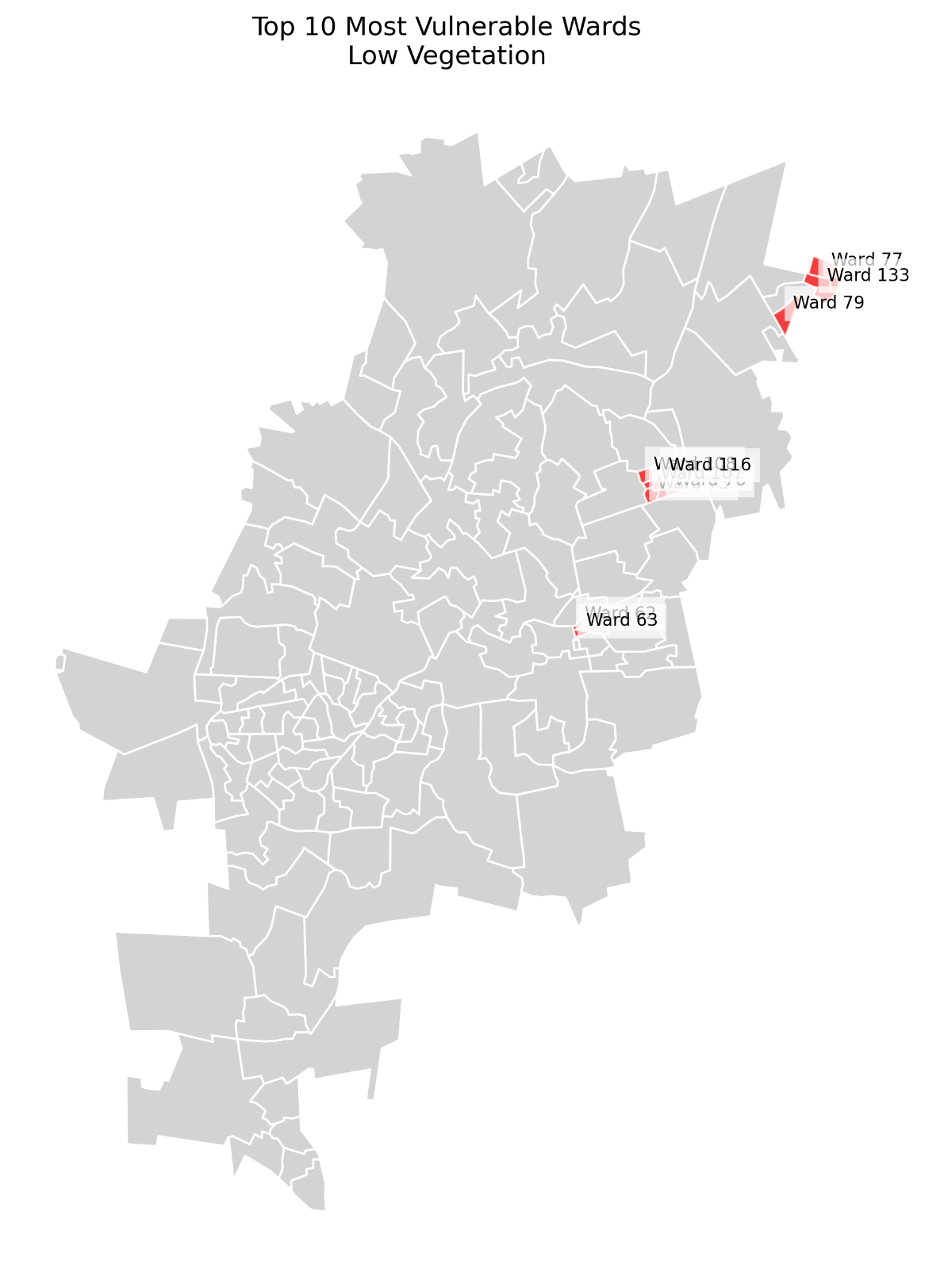

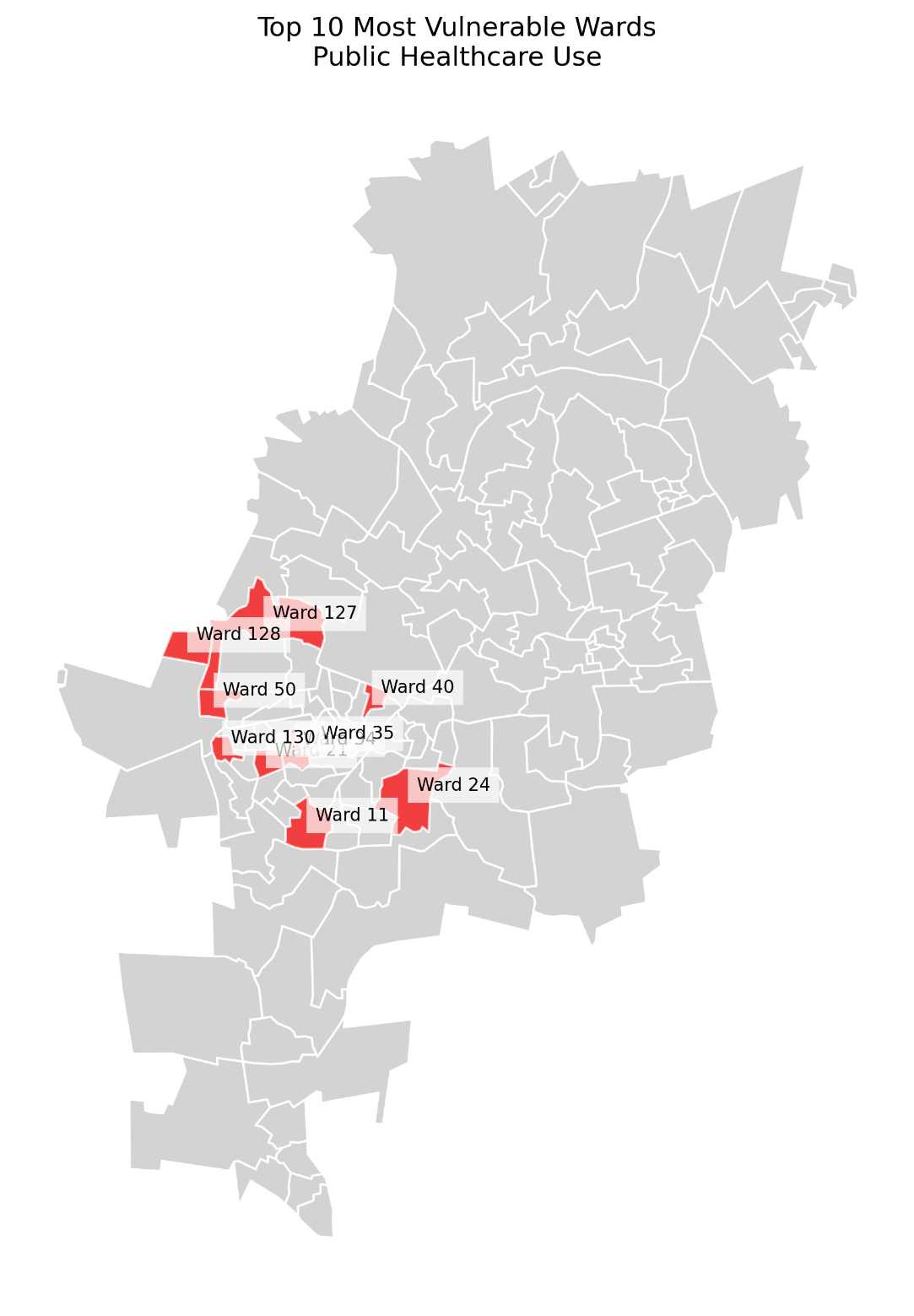

Supplement: Supplementary file 1 — Supplementary file1 (DOCX 1165 KB) [file 484_2025_2971_MOESM1_ESM.docx]
